# *CPT1A, CYGB,* and * MRPL3* in pre-eclampsia: biomarkers identified from oxidative stress-related gene analysis

**DOI:** 10.7717/peerj.21572

**Published:** 2026-07-30

**Authors:** Ling Chen, Meiting Wu

**Affiliations:** Department of Gynecology and Obstetrics, Fujian Medical University Union Hospital, Fuzhou, China

**Keywords:** Pre-eclampsia, Bioinformatics, Oxidative stress, Carnitine palmitoyltransferase 1A

## Abstract

**Background:**

The involvement of oxidative stress (OS) in pre-eclampsia (PE) has been reported, and the present study probed into the OS-related feature genes in PE.

**Methods:**

The dataset GSE60438 was used to identify the OS-related features in PE, and corresponding feature genes were identified using machine learning algorithms including weighted gene co-expression network analysis (WGCNA), Least Absolute Shrinkage and Selection Operator (LASSO) regression analysis and support vector machine-recursive feature elimination (SVM-RFE). The diagnostic value of the feature genes and their correlation with immune infiltration were explored. Gene set enrichment analysis (GSEA) was performed to identify enriched pathways of these genes. Furthermore, regulatory networks involving the feature genes were constructed. The role of these genes in hypoxia/reoxygenation (H/R)-induced trophoblasts was investigated.

**Results:**

Two WGCNA-identified modules (MEpurple and MEturquoise) were intersected with OS-related genes, yielding 359 common genes. Through machine learning algorithms and expression validation, three genes (*CPT1A*, *CYGB* and *MRPL3*) were identified and used to establish a diagnostic model, which reached an AUC of 0.930. The transcription factor (TF) SPI1 and multiple miRNAs formed a potential regulatory network for * CPT1A*, *CYGB* and *MRPL3*, and the expression of these three genes was closely associated with the infiltration of various immune cells (such as monocytes and macrophages M1) in PE. Cellular experiments confirmed that * CPT1A* silencing alleviated trophoblast dysfunction, reduced reactive oxygen species (ROS) levels, and decreased apoptosis under H/R conditions.

**Conclusion:**

The present study examined the OS features in PE, hoping to contribute to the management of PE.

## Introduction

As a major cause of maternal and neonatal morbidity and mortality, pre-eclampsia (PE) is characterized by elevated blood pressure, proteinuria and edema (Dimitriadis et al., 2023; Montgomery et al., 2024; Guo et al., 2023). Advances in the prediction and prophylaxis of PE has contributed to the gradual evolution of this field worldwide (Kornacki et al., 2023). Current management of PE focuses on the alleviation of maternal symptoms and prolonging pregnancy fetal growth as long as possible, thereby reducing complications associated with iatrogenic premature delivery (Zhang et al., 2022). However, in low- and middle-income countries, where PE is prevalent and represents a major contributor to maternal mortality, using biomarkers for predicting PE are difficult to implement due to the scarcity of screening tools (Amelia et al., 2025). Furthermore, despite progress in the development of screening options, further work is needed before a screening test can be clinically implemented to predict the onset of PE (MacDonald et al., 2022).

During the progression of PE, increased oxidative stress (OS) triggers the production of lipid peroxides and reactive oxygen species (ROS), leading to placental vascular endothelial dysfunction and ultimately contributes to PE development (Ahmad, Zimmerman & Moore, 2019). In addition to placental vascular endothelial dysfunction, OS can alter placental remodeling and cause ischemia/reperfusion (I/R) injury with an elevated xanthine oxidase activity (San Juan-Reyes et al., 2020). Therefore, targeting OS may represent a potential therapeutic method to improve both short-term and long-term maternal and fetal outcomes in patients with PE (Afrose, Alfonso-Sánchez & McClements, 2025). At present, a better and more detailed understanding of the mechanisms of action of proposed anti-OS strategies is critical for identifying companion biomarkers and personalized medicine approaches to developing effective treatments of PE.

Driven by interest in identifying gene expression profiles, largely based on microarray data for diagnosis and risk prediction, one study suggested that PE may be a complicated condition influenced by maternal genes (Vennou et al., 2020). Another study employed sequencing data to identify key genes involved in the onset of PE (Li et al., 2024b). It is well recognized that public databases provide ample data for re-analysis and re-evaluation, thereby enhancing the utilization of available resources for more robust analyses (Clough et al., 2024; Egorova et al., 2025). Additionally, machine learning algorithms, particularly feature selection technique, are highly effective in revealing crucial information from high-throughput genomic data, thereby facilitating the identification of crucial genes involved in disease progression (Auslander, Gussow & Koonin, 2021; Cho & Kang, 2020; Shang et al., 2024; Zhang et al., 2024; Zhang et al., 2026). The present study aimed to analyze and screen feature genes related to OS in PE using relevant data from Gene Expression Omnibus (GEO) database (He et al., 2024), applying algorithms including weighted gene co-expression network analysis (WGCNA) and machine learning approaches. Subsequently, functional enrichment analysis and immune infiltration analyses were performed to elucidate the significance of these feature genes in PE. Further experimental assays were conducted to investigate their implication in PE.

## Analytical methods

### Data source

The dataset GSE60438 (Transcriptome profiling of deciduas from pre-eclamptic and normotensive pregnancies) was downloaded from GEO database (Yong et al., 2015). Two datasets WG6v3 (the training set) and HT12v4 (the validation set) were obtained. Specifically, the WG6v3 dataset comprised 23 cases of normal basal decidua samples and 25 cases of basal decidua samples from PE patients, and the HT12v4 dataset comprised 42 normal basal decidua samples and 35 basal decidua samples from PE patients. Additionally, a total of 1,405 OS-related genes were retrieved from GeneCards database (https://www.genecards.org/) based on the correlation score, with the top 10% genes selected for further analysis.

### Identifying feature gene modules by WGCNA

The R package “WGCNA” was applied to identify feature genes modules associated with OS in PE using the training set data (Langfelder & Horvath, 2008). Prior to network construction, the expression matrix was preprocessed to remove outlier samples and genes with low expression in order to improve the reliability of the analysis. The pickSoftThreshold function was subsequently applied to evaluate the scale-free topology fit across a range of candidate soft-thresholding powers. The minimum power achieving a scale-free topology fit index of 0.85 was selected as the soft threshold β for network construction. An adjacency matrix was computed using the chosen β and subsequently transformed into a topological overlap matrix (TOM). Genes were clustered based on the TOM dissimilarity, and modules were identified using the dynamic tree-cut method, with the minimum module size set to 200. Modules whose eigengenes showed high similarity were merged using a mergeCutHeight = 0.2. Finally, the correlation between each module eigengene and the clinical phenotype was calculated, and modules satisfying —cor— ≥ 0.3 with *P* < 0.05 were retained as candidate key modules for downstream analysis (Li et al., 2024a).

### Machine learning algorithms to reveal feature genes

Genes from the WGCNA-identified modules of interest were intersected with OS-related genes from GeneCards database. The overlapping genes were then subjected to Least Absolute Shrinkage and Selection Operator (LASSO) regression analysis using the R package “glmnet” (Engebretsen & Bohlin, 2019). Additionally, these genes were analyzed by the support vector machine-recursive feature elimination (SVM-RFE) algorithm using the R package “e1071” (Kebede, Le Cornet & Fortner, 2023). The results were selected based on the minimum error criterion, the overlapping genes identified by both algorithms were designated as key genes for further analyses.

### Evaluation of expression and the diagnostic potential of the key genes in PE

Box plots were plotted to display the expression of the key genes in the samples of both training set and validation set, and the diagnostic potential of these genes was explored by developing a nomogram using the R package “rms” (Wang et al., 2024). The corresponding ROC curve, AUC value, calibration curve and decision curves were generated or calculated to evaluate the predictive potential and reliability of the nomogram.

### Immune infiltration analysis

Immune infiltration of 22 types of immune cells was analyzed using the R package “CIBERSORT” based on data from the website of CIBERSORT (https://cibersortx.stanford.edu/) (Craven, Gökmen-Polar & Badve, 2021). The immune infiltration profile was compared between sample groups, and the correlation between key genes and immune infiltration was further assessed and visualized using a heatmap.

### Gene set enrichment analysis (GSEA)

PE samples were divided into low- and high-expression groups by the median of the key genes. GSEA was performed using the GSEA software (version 4.4.0) (Wu et al., 2020) based on the background gene sets c2.cp.kegg_medicus and c5.go to elucidate the functional roles of the key genes in PE.

### Plotting the regulatory networks incorporating the key genes

The potential transcription factors (TFs) modulating the key genes in the placenta tissues were predicted using the database hTFtarget (https://guolab.wchscu.cn/hTFtarget/#!/). Additionally, miRNAs targeting the key genes were obtained from the ENCORI database (https://rnasysu.com/encori/). The TFs and miRNAs validated in at least three studies were taken and the corresponding regulatory networks were constructed (He et al., 2025).

### Experimental validation

Cell culture and hypoxia/reoxygenation (H/R) modeling: Roswell Park Memorial Institute-1640 medium (C3250, Solarbio, Beijing, China) added with 5% fetal bovine serum (S9020, Solarbio, China) was used to culture human trophoblasts cell line HTR8/SVneo (CRL-3271, ATCC, Manassas, VA) in the incubator with 5% CO_2_ at 37 °C. STR identification was performed on the cells, and the mycoplasma detection results were negative. For the modeling process, HTR8/SVneo cells were exposed to hypoxic conditions (2% O_2_) at 37 °C for 8 h to induce hypoxia, followed by reoxygenation for 16 h under normoxic conditions (5% CO_2_ in air) (Wang et al., 2025).

Cell liposome transfection: To knockdown *CPT1A* in HTR8/SVneo cells, small interfering RNA (siRNA) against CPT1A (si-*CPT1A* #1, sequence: 5′-GCCATGAAGCT CTTAGACAAA-3′; si-*CPT1A* #2, sequence: 5′-CGTAGCCTTTGGTAAAGGAAT-3′) were synthesized and ordered from Sangon Biotech (Shanghai, China). Cells transfected with the scramble control siRNA were considered as the controls. Transfections were performed using lipofectamine 2000 transfection reagent (11668-027; Invitrogen, Carlsbad, CA, USA) according to the instructions. Cells were harvested 48 h later, and transfection efficiency was tested.

Quantitative real-time PCR assay: TriZol reagent (15596-026; Invitrogen) was utilized to extract total cellular RNA from HTR8/SVneo cells following the manual, and RNA concentration was quantified in NanoDrop 2000 spectrophotometer (ND-2000; Thermo Fisher Scientific, Waltham, MA, USA). Following the synthesis of cDNA using the first-strand cDNA synthesis kit (K16325; Solarbio), quantitative PCR was performed in CFX384 touch real-time PCR system (Bio-Rad Laboratories, Inc., Hercules, CA, USA) using the SYBR Greem qPCR mix (SR1110; Solarbio). With *GAPDH* as a normalization control, the relative mRNA level was finally quantified using the 2^−ΔΔCt^ method (Livak & Schmittgen, 2001). Primers were listed in Table 1.

**Table 1:** Sequences of primers for the PCR assay.

Gene	Primers (5′–3′)	
	Forward	Reverse
CPT1A	GATCCTGGACAATACCTCGGAG	CTCCACAGCATCAAGAGACTGC
GAPDH	GTCTCCTCTGACTTCAACAGCG	ACCACCCTGTTGCTGTAGCCAA

Cell viability assay: Following the liposome transfection and H/R modeling, HTR-8/SVneo cells at 1 × 10^4^ cells/well were planted into 96-well plates. After cell incubation for 24, 48 and 72 h at 37 °C, CCK-8 working solution (CA1210; Solarbio) was supplemented for another 1-h incubation at 37 °C. The optical density was recorded at 450 nm with a microplate reader (Bio-Rad Laboratories, Inc.).

Cell migration assay: In detail, HTR-8/SVneo cells were seeded into 6-well plates at 1 × 10^5^ cells/well until 90% confluence. Thereafter, a straight wound on monolayer was made with a sterile 100 µL pipette tip. Next, the cells were incubated in serum-free medium for 48 h. The gap area was imaged using an inverted optical microscope (Olympus, Tokyo, Japan), and the wound closure was analyzed by ImageJ software (version 1.48v; National Institutes of Health, Bethesda, MD, USA).

Cell invasion assay: A total of 5 × 10^4^ HTR-8/SVneo cells were suspended in 200 µL serum-free medium and plated into the upper chambers of Transwell inserts (pore size: 8 µm; #3422, Corning, Inc., Corning, NY, USA) coated with Matrigel (356234, BD Biosciences, Franklin Lakes, NJ, USA) at 37 °C for 1 h. The lower chambers contained normal culture medium with 10% serum. Following 48-h cell culture at 37 °C, the invaded cells were fixed with 4% paraformaldehyde (P1110; Solarbio) for 20 min at 37 °C and colored with 0.1% crystal violet (C8470; Solarbio) for 10 min at ambient temperature. The images were obtained under an inverted optical microscope (Olympus, Japan), and the invading cells were quantified using ImageJ software.

Quantification on the ROS level: Cellular ROS level was measured as previously described (Chen, Wu & Zhou, 2024). Briefly, cells were stained with 10 µM 2,7-dichlorodihydrofluorescein diacetate (DCFH-DA) (D6470; Solarbio) for 20 min at 37 °C in the dark. ROS level was then analyzed with the CytoFLEX LX flow cytometer (Beckman Coulter, Indianapolis, IN, USA), and the mean fluorescence intensity (MFI) was recorded.

Cell apoptosis assay: using a commercial kit (CA1020; Solarbio), apoptosis of HTR8/SVneo cells was determined *via* flow cytometry. After centrifugation, the supernatant was removed and cells were subsequently added with the working solution of Annexin V-FITC and propidium iodide for 20-min incubation at room temperature in the dark. CytoFLEX LX flow cytometer and FlowJo software (v. 11; FlowJo, LLC., Ashland, OR) were employed for final analysis.

### Statistical analyses

Computational analyses were performed in R software (version 3.6.0; R Core Team, 2019) unless specified, and the laboratory data analyses were performed using GraphPad Prism software (version 8.0.2). Unpaired *t*-test was used for two-group comparisons. One-way ANOVA followed by Tukey’s multiple comparisons test and two-way analysis of variance (ANOVA) followed by Šídák’s multiple comparisons test were applied where appropriate. All experiments were repeated at least three times, and data were expressed as mean ± standard deviation (SD). A *p* < 0.05 stood for statistical significance.

## Results

### Identification of PE-relevant gene modules *via* WGCNA

**Figure 1:**
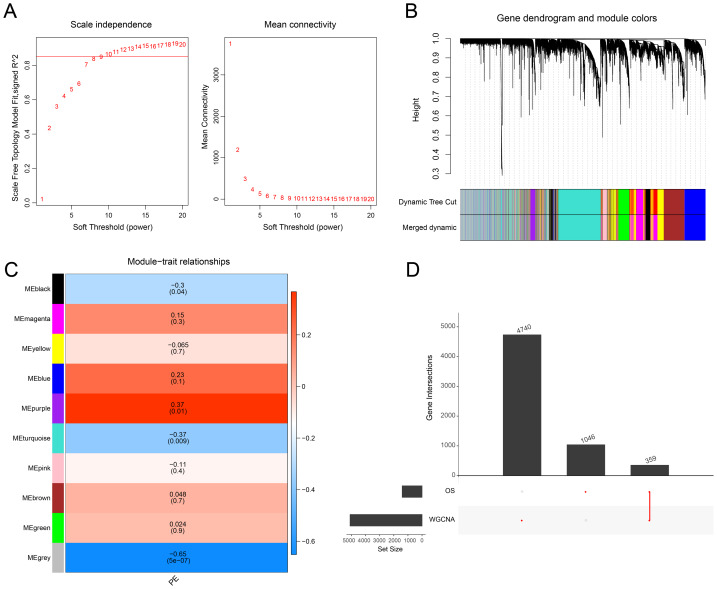
Identification of pre-eclampsia-relevant gene modules *via* WGCNA. (A) Soft-threshold power determination; (B) closely connected genes in the key modules in cluster dendrogram. (C) Relationships between the identified gene modules and PE based on the coefficients and *p*-values. (D) Identification of key genes from the gene modules MEpurple and MEturquoise and OS-correlated genes from GeneCards database.

Data from the training set (WG6v3 data of dataset GSE60438) were analyzed by WGCNA to identify gene modules. First, by evaluating the scale-free topological fit index across a range of soft thresholds, the optimal soft threshold (β) for network construction was 10, at which the network structure conformed to a scale-free topology (Fig. 1A). A gene clustering dendrogram based on the topological overlap matrix was generated, and 10 co-expressed gene modules were identified using the dynamic tree-cut method, each assigned a distinct color (Fig. 1B). Additionally, Fig. 1C presents a heatmap of module-phenotype correlations. The MEpurple module (correlation coefficient: 0.37, *P* = 0.01) was significantly positively correlated with PE, while the MEturquoise module was negatively linked to PE (correlation coefficient: −0.37, *P* = 0.09). Therefore, these modules were selected as feature modules for subsequent analysis. Figure 1D shows the intersection of genes from these two modules with OS-correlated genes, yielding 359 overlapping genes. This result provided the basis for subsequent screening of key genes using machine learning algorithms.

### Machine learning algorithms on revealing the key genes

**Figure 2:**
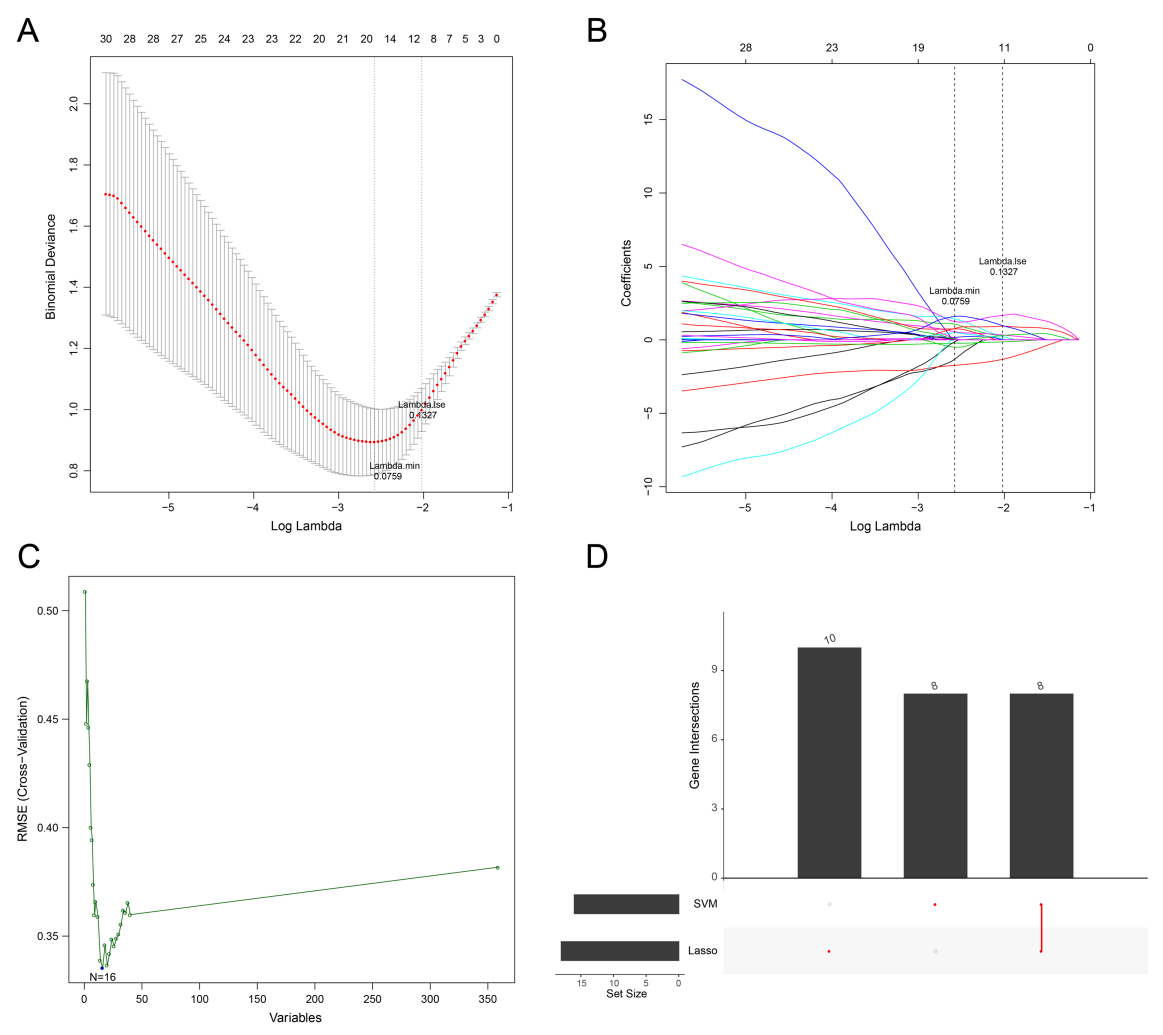
Machine learning algorithms on revealing the key genes related to pre-eclampsia. (A–B) LASSO regression analysis was applied as a machine learning algorithm to reveal the key genes related to pre-eclampsia. (C) SVM-RFE analysis was another machine learning algorithm to reveal the key genes related to pre-eclampsia. (D) Identification of the common genes related to both LASSO regression analysis and SVM-RFE analysis.

Thereafter, two machine learning algorithms were applied to further identify key genes from the 359 common genes. Using LASSO regression analysis, 18 feature genes were selected when the error was minimized (lambda.min = 0.0759, Figs. 2A–2B). Based on SVM-RFE analysis, the minimum error was achieved when the number of gene was 16; therefore, 16 feature genes were selected (Fig. 2C). The intersection of the genes identified by the two methods yielded eight key genes: *ATF4*, *SMN1*, *CPT1A*, *MRPL3*, *CYGB*, *HSPD1*, *CLN6* and *HMGB1* (Fig. 2D).

### Validation of the diagnostic potential of the key genes

**Figure 3:**
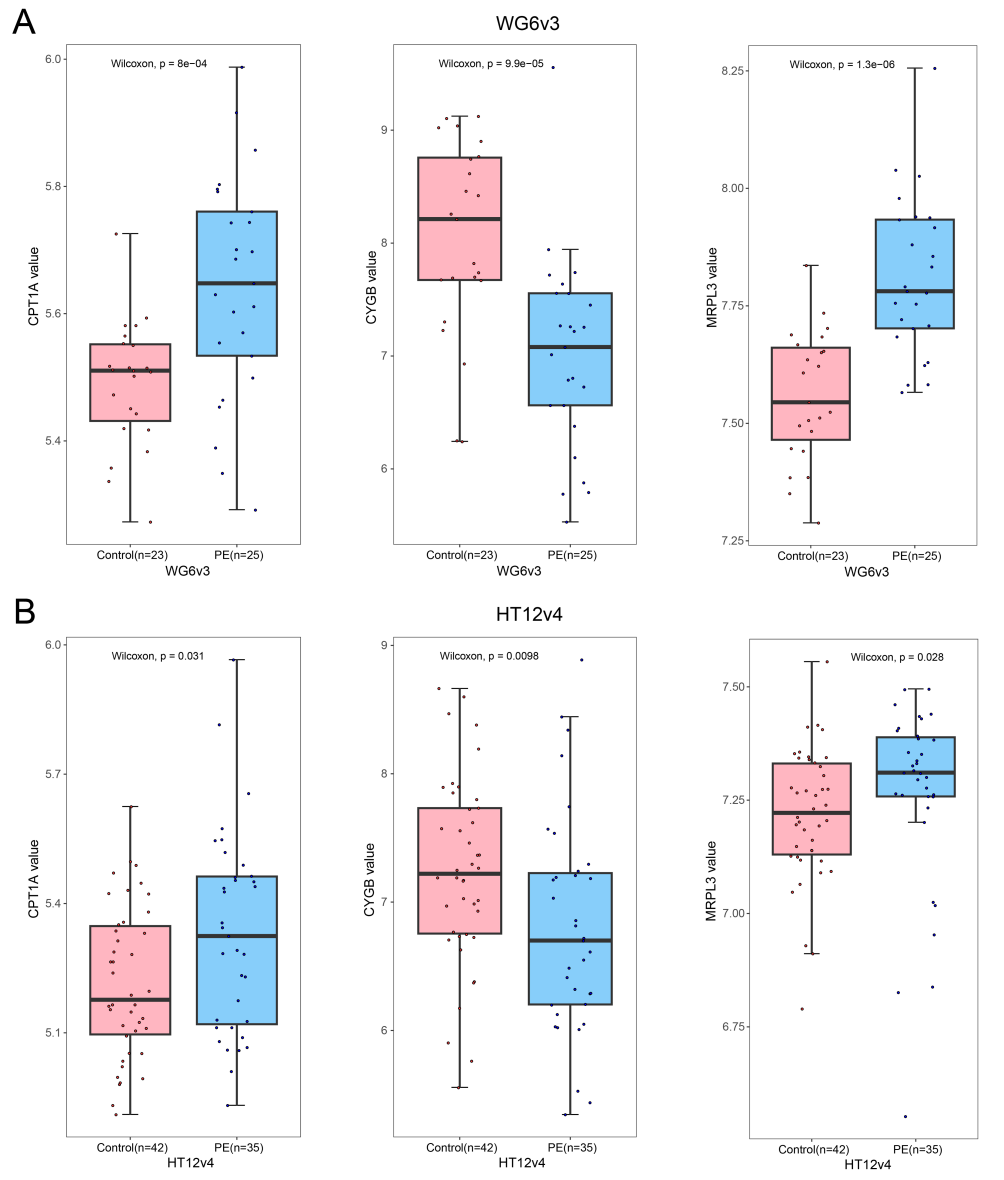
Quantification on the levels of key genes in pre-eclampsia. (A–B) Expression trends of key genes CPT1A, CYGB, and MRPL3 in the placenta samples of pre-eclampsia patients and healthy controls of both the training set WG6v3 and validation set HT12v4.

Subsequently, the expression level of these eight genes in the placenta samples of PE patients and healthy controls of both the training set and validation set were quantified and compared (Figs. 3A–3B). Only three genes (*CPT1A*, *MRPL3*, and *CYGB*) showed consistent expression trends across the two datasets and were therefore retained for further analysis. Specifically, in placental samples from PE patients, *CPT1A* and *MRPL3* were significantly upregulated, whereas *CYGB* was significantly downregulated.

**Figure 4:**
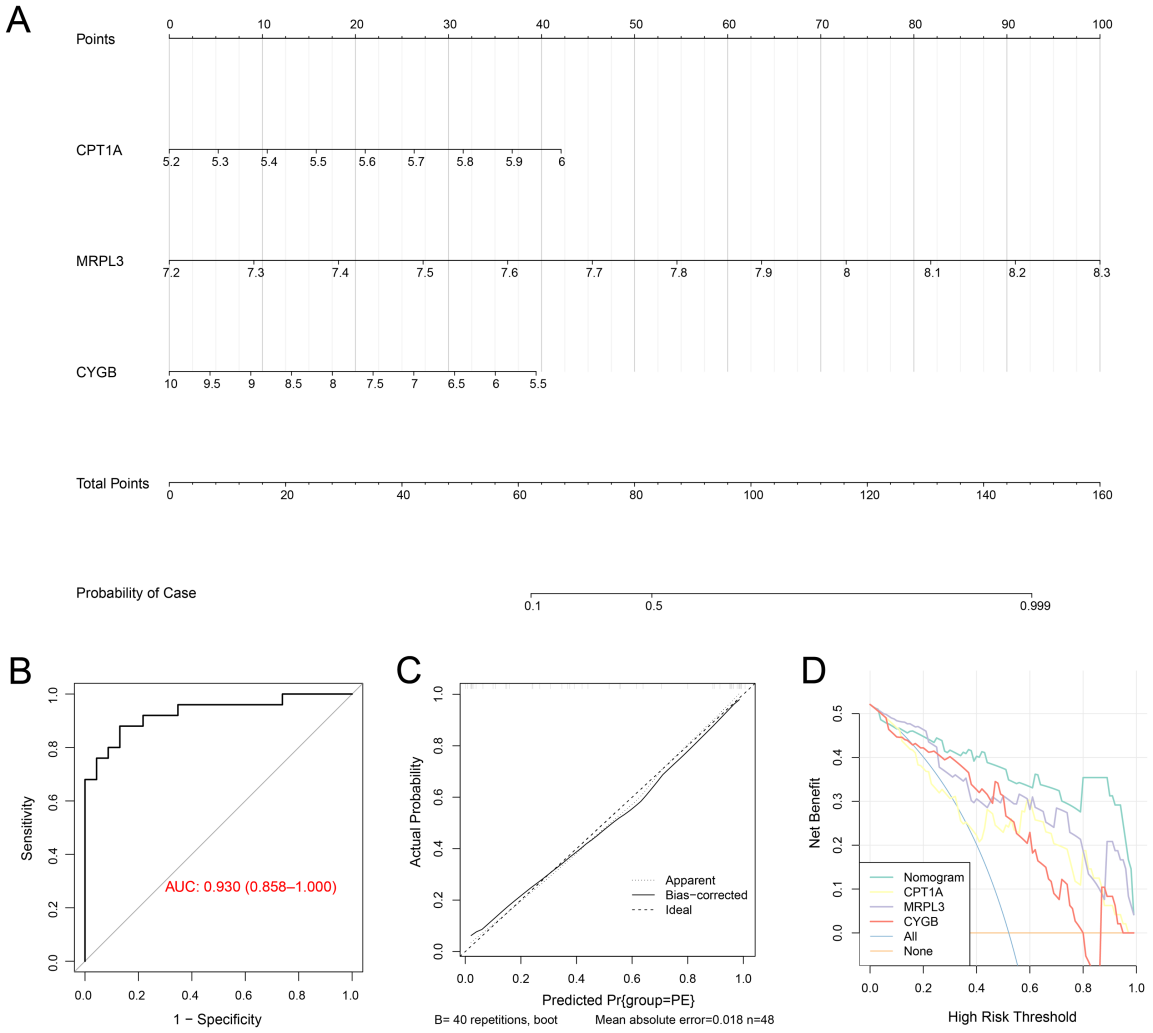
Validation on the diagnosis performance of the three key genes in pre-eclampsia. (A) Nomogram incorporating the level of the three key genes (CPT1A, CYGB, and MRPL3) plotted to explore the diagnostic potential. (B–D) The corresponding ROC curve (B), calibration curve (C) and the decision curve (D) for the nomogram.

A nomogram incorporating the expression level of these three key genes was plotted to explore the diagnostic potential (Fig. 4A), and the corresponding ROC curve demonstrated a strong reliability of the nomogram (Fig. 4B, AUC = 0.93). Additionally, calibration curves and decision curves showed that the calibration curve closely aligned with the actual curve (Fig. 4C) and that the net benefit of the nomogram was evidently higher than any of the single gene (Fig. 4D).

### Correlation of the key genes with immune infiltration

**Figure 5:**
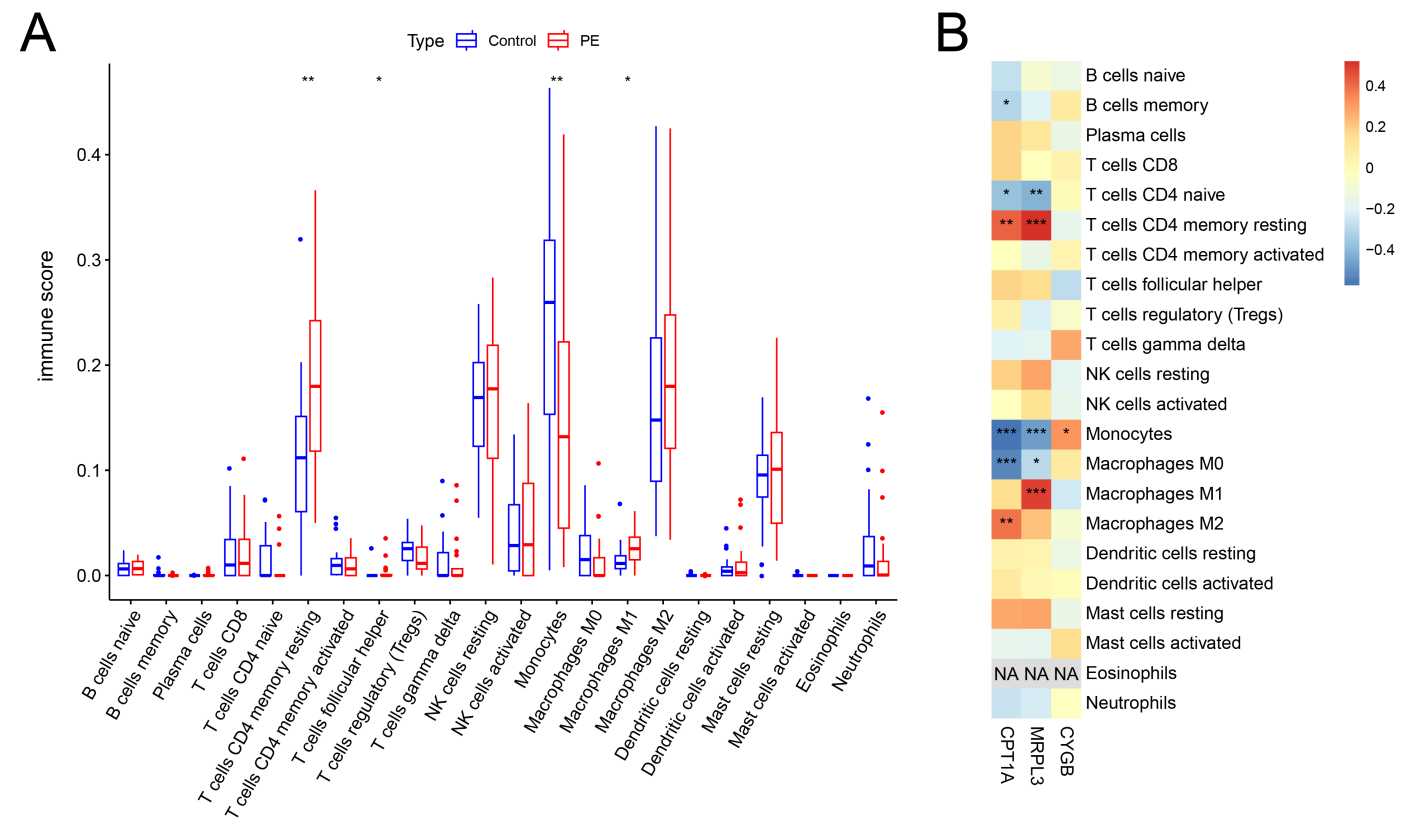
Correlation of the key genes with immune infiltration in pre-eclampsia. (A) Relative abundance of immune cells in the placenta tissue based on the data of CIBERSORT. (B) Correlation of the key genes with immune infiltration in pre-eclampsia. *** *p* < 0.001, ** *p* < 0.01, and * *p* < 0.05.

The immune infiltration in PE was analyzed using the training set. Significant differences were observed in the infiltration of four types of immune cells, including macrophage M1, T cells CD4 memory resting, T cells CD4 memory activated, and monocytes (Fig. 5A). Further correlation analysis revealed distinct correlation patterns. *CPT1A* was positively linked to the infiltration of T cells CD4 memory resting and macrophages M2, but negatively related to that of monocytes and macrophages M0. *MRPL3* was positively correlated with the infiltration of T cells CD4 memory resting and macrophages M1, but negatively linked to that of monocytes and macrophages M0. *CYGB* was positively correlated with the infiltration of monocytes (Fig. 5A).

### GSEA to reveal enriched pathways

**Figure 6:**
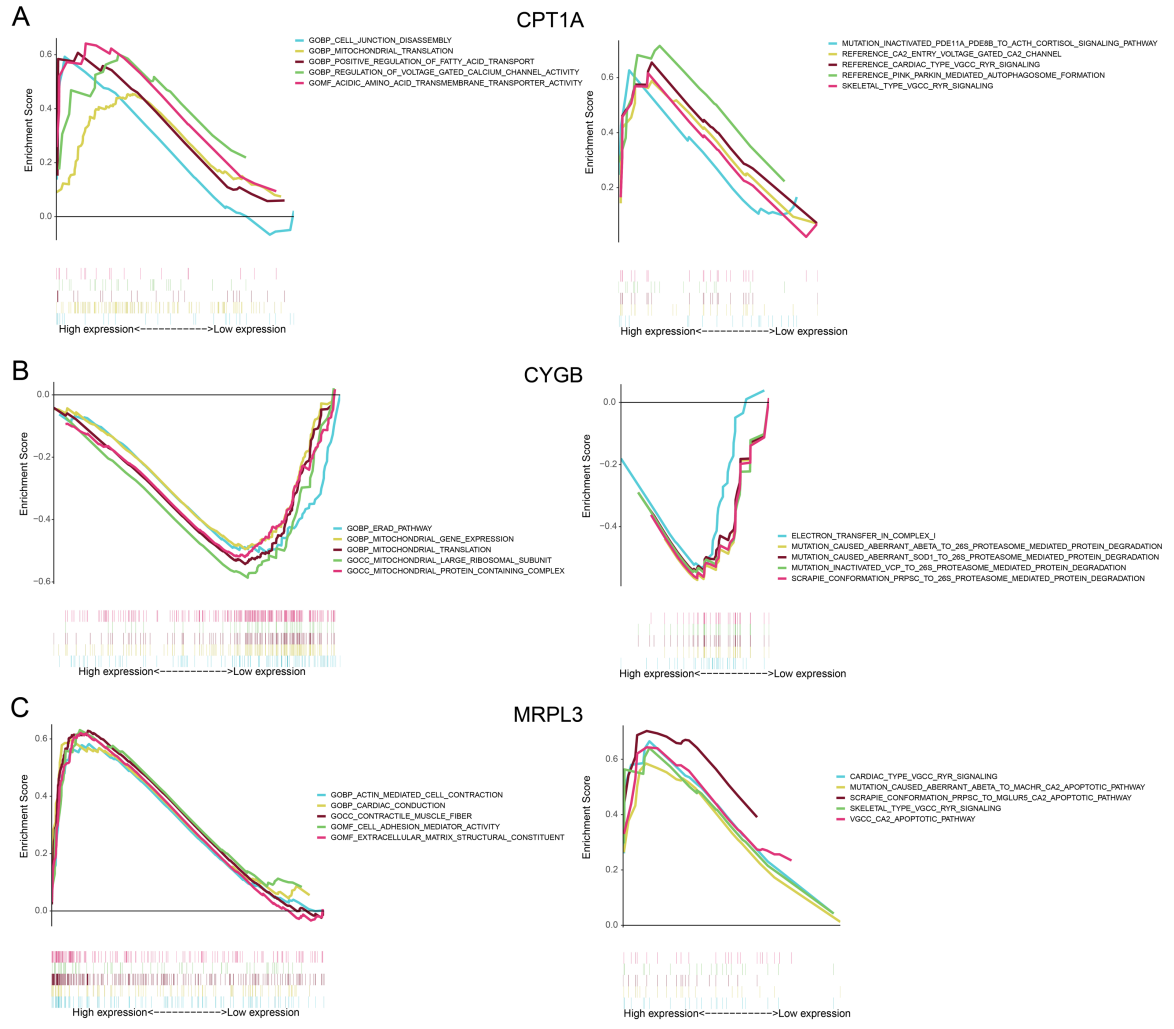
GSEA on revealing the enriched pathways. (A–C) GSEA was performed to reveal the enriched pathways in pre-eclampsia patients with high/low CPT1A, CYGB and MRPL3 expression levels.

GSEA revealed that the three key genes were enriched to mitochondria-related pathways including mitochondrial translation, mitochondrial protein containing complex, and mitochondrial gene expression, along with VGCC CA2 apoptotic pathway (Figs. 6A–6C).

### Plotting the regulatory network based on the key genes

**Figure 7:**
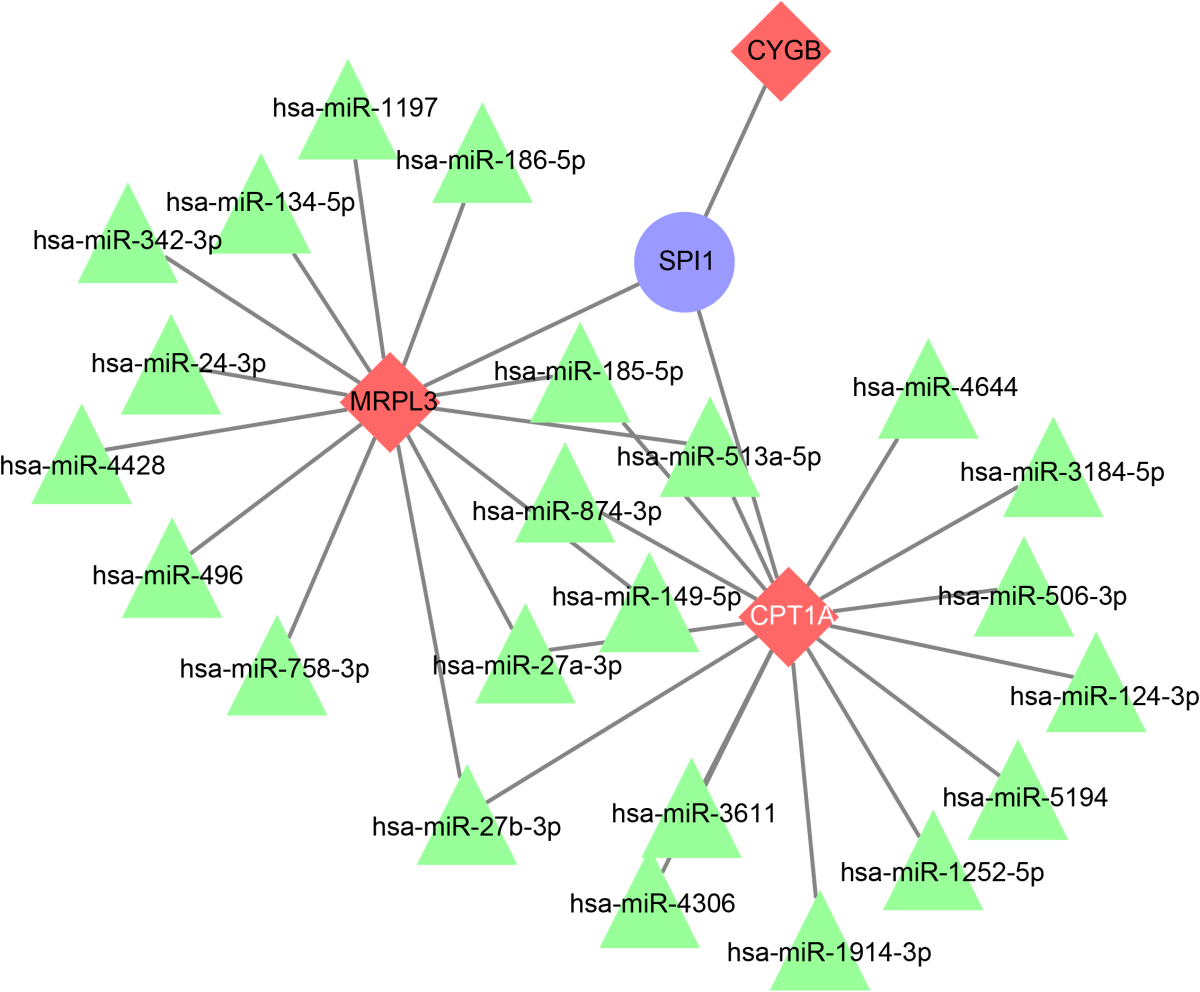
Plotting of the regulatory network based on the relevant transcription factors and miRNAs. The regulatory network was plotted based on the transcription factors which may regulate the key genes in the placenta from hTFtarget prediction and the miRNAs from the ENCORI database.

Additionally, the hTFtarget database was applied to identify the common TF(s) regulating the three genes, and SPI1 was considered as the common TF. According to the ENCORI database, 14 and 13 miRNAs that potentially targeted *CPT1A* and *MRPL3*, respectively, with each targeting relationship validated in at least three studies, with each targeting relationship validated in at least three studies. In contrast, no miRNA targeting *CYGB* met the selection criteria. These regulatory relationship were uploaded into the cytoscape software to construct the regulatory network (Fig. 7).

### Experimental validation on the involvement of *CPT1A* in H/R-modeled trophoblasts

**Figure 8:**
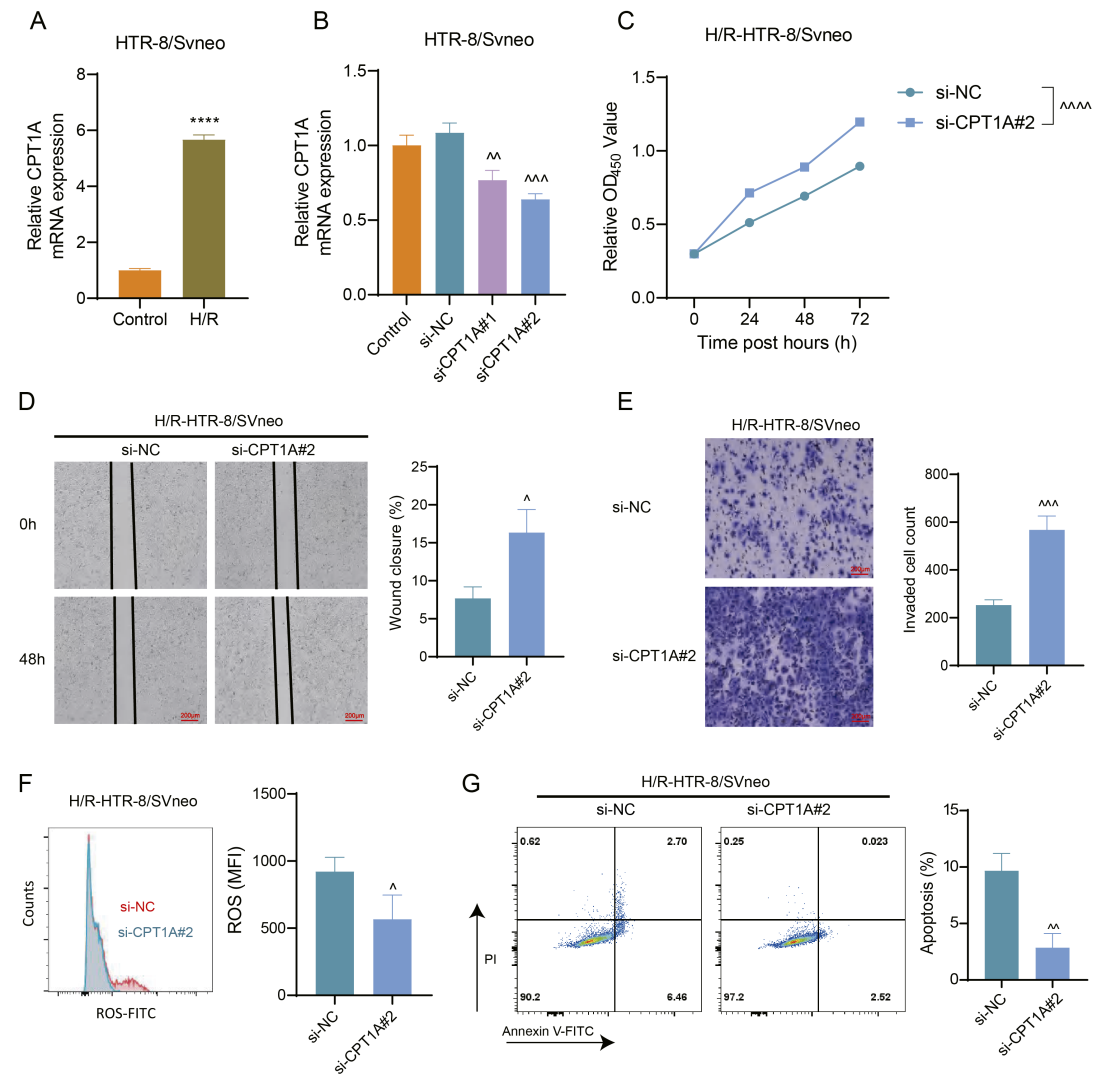
Validation on the effects of CPT1A on hypoxia/reoxygenation-induced trophoblasts HTR8/SVneo. (A) The quantified CPT1A mRNA expression level in HTR8/SVneo cells with/out hypoxia/reoxygenation modeling. (B) Verification on the knockdown efficiency of CPT1A-specific small interfering RNAs. (C) The effects of CPT1A silencing on the viability of HTR8/SVneo cells following hypoxia/reoxygenation modeling detected by CCK-8 assay. (D) The migration of HTR8/SVneo cells following hypoxia/reoxygenation modeling detected by scratch assay. (E) The invasion of HTR8/SVneo cells following hypoxia/reoxygenation modeling detected by Transwell assay. (F) The ROS content of HTR8/SVneo cells following hypoxia/reoxygenation modeling. (G) The results of flow cytometry assay on the apoptosis of HTR8/SVneo cells following hypoxia/reoxygenation modeling. All procedures were performed in triplicate (*n* = 3). * *vs.* Control; ˆ*vs.* si-NC. ˆ*p* < 0.05, ˆˆ*p* < 0.01, ˆˆˆ*p* < 0.001, and **** *p* or ˆˆˆˆ*p* < 0.0001.

A series of laboratory assays were performed to validate the effects of *CPT1A* in H/R-modeled trophoblasts. An elevated level of *CPT1A* was observed in the modeled trophoblasts (Fig. 8A), and the corresponding knockdown assays were applied lower CPT1A expression (Fig. 8B). Furthermore, CCK-8, scratch and Transwell assays demonstrated that *CPT1A* silencing could promote the survival, migration and invasion of H/R-modeled trophoblasts (Figs. 8C–8E). Flow cytometry also showed that silencing *CPT1A* visibly reduced the ROS content and apoptosis of H/R-modeled trophoblasts (Figs. 8F–8G).

## Discussion

Although increasing attention has been paid to PE, no effective treatment for PE is currently available. Symptoms of PE can generally be alleviated only after placental delivery, which has therefore highlighted the importance of prediction and prevention (Roberts, 2024). In our *in-silico* analysis using WGCNA and the two machine learning algorithms identified three PE-related genes (*CPT1A*, *MRPL3* and *CYGB*). Subsequently, a nomogram incorporating these three genes was created, showing a strong diagnostic potential for PE. Further cellular validation assays demonstrated an elevated level of *CPT1A* in the H/R-modeled trophoblasts, and that silencing *CPT1A* promoted the survival, migration and invasion yet reduced the ROS content and apoptosis of modeled trophoblasts. These data therefore underscored the therapeutic potential of the three genes in PE.

Several previous reviews have revealed the mechanisms of physiopathogenesis of PE, including OS (San Juan-Reyes et al., 2020). In the placenta, OS is believed to arise from disruption of the balance between ROS production and detoxification (Wu et al., 2025). Subsequently, the by-products trigger inflammatory responses and are detrimental to cells, thereby leading to premature placental aging. Such placental prematurity may result in reduced functional capacity and abnormal pregnancy outcomes such as PE (Joo et al., 2021). Studies have used the data of GEO database to investigate genes implicated in PE progression and detection, with special attention paid to the relationship between OS and PE (Cao et al., 2024; Guo, Li & Xiong, 2025; Hamdan, 2025; Yu et al., 2025). Integration of data from GEO can increase sample size in a cost-effective manner; however, meticulous attention should be paid to methodological details to avoid compromising results. In the present study, the dataset GSE60438 was used to identify OS-related genes in PE, leading to the identification of three key genes (*CPT1A*, *MRPL3*, and *CYGB*) for subsequent analysis. The carnitine palmitoyltransferases family member *CPT1A* is a rate-limiting enzyme for fatty acid β-oxidation, and its inhibition has been demonstrated to reduce OS-induced chondrocyte senescence (Schlaepfer & Joshi, 2020; Jiang et al., 2022). Notably, our computational analysis revealed that *CPT1A* expression was elevated in PE placental tissues, a finding that aligns with recent experimental evidence demonstrating *CPT1A* upregulation in PE placentas and its role in promoting PE progression *via* the PI3K/AKT/mTOR pathway (Chen et al., 2023). *MRPL3* is a mitochondrial ribosomal protein encoded by the nuclear genome and transported into mitochondria for ribosome biogenesis (Ahmad et al., 2025). *CYGB* is predominantly expressed in hepatic stellate cells and plays a critical role in attenuating mitochondrial OS (Okina et al., 2023). Concerning their association to PE, previous studies observed increased levels of *CYGB* in the serum of PE patients (Tayyar et al., 2019), while we detected reduced levels of *CYGB* in PE tissue. This discrepancy may reflect tissue-specific expression patterns or distinct regulatory mechanisms. The diagnostic model incorporating these three genes demonstrated strong diagnostic potential for PE. Furthermore, our cellular validation assays validated that *CPT1A* was upregulated in H/R-modeled trophoblasts, and that silencing *CPT1A* promoted cell survival, migration, and invasion while reducing ROS content and apoptosis. These functional observations were consistent with our computational findings of *CPT1A* overexpression in PE tissues and corroborate recent reports that *CPT1A* knockdown enhances trophoblast invasion and migration (Chen et al., 2023). Moreover, *CPT1A*-mediated fatty acid oxidation has been implicated in endothelial cell function and angiogenesis in PE (Wu et al., 2024), further supporting its pathogenic role. All these results suggest a direct connection between *CPT1A* overexpression in PE tissues and its functional influence on trophoblast behavior. Future studies are warranted to clarify the mechanism of the other two genes related to PE.

Immune cells function crucially in supporting embryo implantation and developing maternal-fetal interface. In PE development, immune dysfunction and inflammation have been recognized as the key risk factors (Deer et al., 2023). Disruption of the complex immune microenvironment during pregnancy can lead to pro-inflammatory reactions, OS, and endothelial dysfunction, thereby potentially exciting PE and adverse outcomes (Aneman et al., 2020; Opichka et al., 2021). Based on the data of CIBERSORT, our current study unveiled significant difference in the infiltration of monocytes, T cells CD4 memory, macrophage M1. Additionally, correlation analysis between the key genes and immune infiltration revealed distinct patterns. Specifically, *CPT1A* was positively linked to the infiltration of T cells CD4 memory resting and macrophages M2, but yet negatively linked to that of monocytes and macrophages M0. *MRPL3* was positively linked to the infiltration of T cells CD4 memory resting and macrophages M1, but negatively linked to that of monocytes and macrophages M0. *CYGB* was positively correlated with the infiltration of monocytes. Previous studies have unveiled the lower activation of memory CD4^+^ T cell subsets in and after PE, whereas altered functional activity of monocyte-macrophage system, key component of innate immunity, suggests a dysfunctional maternal immune response (Kieffer et al., 2019; Vishnyakova et al., 2019). These findings suggest a crucial role for these identified genes in immune dysfunction associated with PE.

Our GSEA revealed that the three genes were enriched to several mitochondria-related pathways including mitochondrial gene expression, mitochondrial translation, mitochondrial protein containing complex, and VGCC CA2 apoptotic pathway. Notably, mitochondrial dysfunction has been observed in the pathogenesis of PE (Smith et al., 2021; Hu & Zhang, 2022). In particular, in PE placentas, mitochondrial fission mediated by hypoxia may activate mitophagy machinery, thereby promoting mitochondrial fragmentation and damages to placental tissues (Kobayashi et al., 2024). The mitochondrial calcium overload, additionally, has been considered as a pro-apoptotic pathway that induces the swelling of mitochondria (Giorgi et al., 2012). However, calcium deficiency in women is related to a higher PE risk (Lu et al., 2024). These findings suggest potential biological processes underlying PE and provided a reference for the development of management strategies.

Nonetheless, there are some limitations that should be addressed. First, the screening of key genes is primarily based on a single public database and specific machine learning algorithms, which may introduce dataset-specific and algorithmic biases. We plan to integrate multiple independent datasets and employ diverse computational methods for cross-validation to enhance the robustness and generalizability of the screening results. Second, the diagnostic model was evaluated only using the training set and has not been validated using an independent external dataset. We plan to collect multicenter clinical samples and utilize additional public data resources to for external validation to objective assess the model’s generalizability. Third, although transcription factors and miRNAs regulating *CPT1A*, *MRPL3*, and *CYGB* were predicted using computational analyses, their regulatory relationships have not yet been experimentally validated. In the future, we plan to use techniques such as dual-luciferase reporter assays and chromatin immunoprecipitation to clarify direct interactions among these molecules. Finally, the application of the three identified genes to early pregnancy screening still faces challenges regarding test sensitivity and stability. Future study could integrate these genes with higher-precision techniques, such as single-cell sequencing, and evaluate their clinical value as early predictive biomarkers in prospective cohorts. These improvements are expected to further solidify the scientific value and clinical translation potential of the findings from this study.

## Conclusion

Collectively, the present study revealed three OS-related key genes (*CPT1A*, *MRPL3* and *CYGB*) and unraveled their diagnostic potential and association with the immune microenvironment in PE. Further explanation using H/R-modeled trophoblasts has demonstrated the potential involvement of *CPT1A* in PE. These findings further provided data for the development of relevant prevention and management strategies for PE.

## Supplemental Information

10.7717/peerj.21572/supp-1 Supplemental Information 1 MIQE checklist

## Abbreviations

PE: Pre-eclampsia
OS: oxidative stress
ROS: reactive oxygen species
I/R: ischemia/reperfusion
GEO: gene expression omnibus
WGCNA: weighted gene co-expression network analysis
SVM-RFE: support vector machine-recursive feature elimination
ROC: receiver operating characteristics
AUC: area under the curve
GSEA: Gene set enrichment analysis
TFs: transcription factors
miRNAs: microRNAs
H/R: hypoxia/reoxygenation
cDNA: complementary DNA
CCK-8: Cell Counting Kit-8
DCFH-DA: 2,7-dichlorodihydrofluorescein diacetate
MFI: mean fluorescence intensity

## Additional Information and Declarations

### Data Availability

The following information was supplied regarding data availability: The data is available at GitHub and Zenodo: - https://github.com/chenling2026/Oxidative-Stress-data
- chenling2026. (2026). chenling2026/Oxidative-Stress-data: Oxidative-Stress-data (Oxidative-Stress-data). Zenodo. https://doi.org/10.5281/zenodo.19366560
The raw sequence data are available at NCBI: GSE60438

## References

[ref-1] Afrose D, Alfonso-Sánchez S, McClements L (2025). Targeting oxidative stress in preeclampsia. Hypertension in Pregnancy.

[ref-2] Ahmad M, Alvi SS, Dhasmana A, Benavidez J, Yallapu MM, Kim DJ, Chauhan SC, Hafeez BB (2025). Mitochondrial ribosomal protein L3 (MRPL3): an early diagnostic biomarker and potential molecular target in pancreatic cancer. Translational Oncology.

[ref-3] Ahmad IM, Zimmerman MC, Moore TA (2019). Oxidative stress in early pregnancy and the risk of preeclampsia. Pregnancy Hypertension.

[ref-4] Amelia M, Siahaan WH, Wijaya N, Harahap AM, Rivany MA, Lumbanraja SN (2025). Pre-eclampsia screening with maternal risk factors and ophthalmic Doppler artery: a systematic review. Hypertension in Pregnancy.

[ref-5] Aneman I, Pienaar D, Suvakov S, Simic TP, Garovic VD, McClements L (2020). Mechanisms of key innate immune cells in early- and late-onset preeclampsia. Frontiers in Immunology.

[ref-6] Auslander N, Gussow AB, Koonin EV (2021). Incorporating machine learning into established bioinformatics frameworks. International Journal of Molecular Sciences.

[ref-7] Cao J, Jiang W, Yin Z, Li N, Tong C, Qi H (2024). Mechanistic study of pre-eclampsia and macrophage-associated molecular networks: bioinformatics insights from multiple datasets. Frontiers in Genetics.

[ref-8] Chen M, Chao B, Xu J, Liu Z, Tao Y, He J, Wang J, Yang H, Luo X, Qi H (2023). CPT1A modulates PI3K/Akt/mTOR pathway to promote preeclampsia. Placenta.

[ref-9] Chen L, Wu M, Zhou Y (2024). HSPB8 binding to c-Myc alleviates hypoxia/reoxygenation-induced trophoblast cell dysfunction. Experimental and Therapeutic Medicine.

[ref-10] Cho YR, Kang M (2020). Interpretable machine learning in bioinformatics. Methods.

[ref-11] Clough E, Barrett T, Wilhite SE, Ledoux P, Evangelista C, Kim IF, Tomashevsky M, Marshall KA, Phillippy KH, Sherman PM, Lee H, Zhang N, Serova N, Wagner L, Zalunin V, Kochergin A, Soboleva A (2024). NCBI GEO: archive for gene expression and epigenomics data sets: 23-year update. Nucleic Acids Research.

[ref-12] Craven KE, Gökmen-Polar Y, Badve SS (2021). CIBERSORT analysis of TCGA and METABRIC identifies subgroups with better outcomes in triple negative breast cancer. Scientific Reports.

[ref-13] Deer E, Herrock O, Campbell N, Cornelius D, Fitzgerald S, Amaral LM, LaMarca B (2023). The role of immune cells and mediators in preeclampsia. Nature Reviews. Nephrology.

[ref-14] Dimitriadis E, Rolnik DL, Zhou W, Estrada-Gutierrez G, Koga K, Francisco RPV, Whitehead C, Hyett J, da Silva Costa F, Nicolaides K, Menkhorst E (2023). Pre-eclampsia. Nature Reviews. Disease Primers.

[ref-15] Egorova D, Olsson B, Kir’yanova T, Plotnikova E (2025). An assessment of the degradation potential and genomic insights towards hydroxylated biphenyls by *Rhodococcus opacus* strain KT112-7. Current Genomics.

[ref-16] Engebretsen S, Bohlin J (2019). Statistical predictions with glmnet. Clinical Epigenetics.

[ref-17] Giorgi C, Baldassari F, Bononi A, Bonora M, De Marchi E, Marchi S, Missiroli S, Patergnani S, Rimessi A, Suski JM, Wieckowski MR, Pinton P (2012). Mitochondrial Ca(2+) and apoptosis. Cell Calcium.

[ref-18] Guo X, Li S, Xiong G (2025). Iron metabolism and preeclampsia: new insights from bioinformatics analysis. The Journal of Maternal-Fetal & Neonatal Medicine.

[ref-19] Guo M, Yan P, Zhu M, Choi M, Li X, Huang J, Zou J, Yuan J, Ding W, Li D, Han X, Wang Y, Wu J (2023). Microcystin-LR prenatal exposure drives preeclampsia-like changes in mice by inhibiting the expression of TGF-β and VEGFA. Food and Chemical Toxicology.

[ref-20] Hamdan HZ (2025). Exploring gene expression signatures in preeclampsia and identifying hub genes through bioinformatic analysis. Placenta.

[ref-21] He L, Shen K, He L, Chen Y, Tang Z (2024). The mechanism of plantaginis semen in the treatment of diabetic nephropathy based on network pharmacology and molecular docking technology. Endocrine, Metabolic & Immune Disorders Drug Targets.

[ref-22] He W, Zhang Q, Xie J, Wu F, Wang L, Wang L (2025). Integrative analyses identify the mechanism by which HSPA9 influences glioma energy metabolism. Oncologie.

[ref-23] Hu XQ, Zhang L (2022). Mitochondrial dysfunction in the pathogenesis of preeclampsia. Current Hypertension Reports.

[ref-24] Jiang N, Xing B, Peng R, Shang J, Wu B, Xiao P, Lin S, Xu X, Lu H (2022). Inhibition of Cpt1a alleviates oxidative stress-induced chondrocyte senescence *via* regulating mitochondrial dysfunction and activating mitophagy. Mechanisms of Ageing and Development.

[ref-25] Joo EH, Kim YR, Kim N, Jung JE, Han SH, Cho HY (2021). Effect of endogenic and exogenic oxidative stress triggers on adverse pregnancy outcomes: preeclampsia, fetal growth restriction, gestational diabetes mellitus and preterm birth. International Journal of Molecular Sciences.

[ref-26] Kebede MM, Le Cornet C, Fortner RT (2023). In-depth evaluation of machine learning methods for semi-automating article screening in a systematic review of mechanistic literature. Research Synthesis Methods.

[ref-27] Kieffer TEC, Scherjon SA, Faas MM, Prins JR (2019). Lower activation of CD4(+) memory T cells in preeclampsia compared to healthy pregnancies persists postpartum. Journal of Reproductive Immunology.

[ref-28] Kobayashi H, Yoshimoto C, Matsubara S, Shigetomi H, Imanaka S (2024). An integral role of mitochondrial function in the pathophysiology of preeclampsia. Molecular Biology Reports.

[ref-29] Kornacki J, Olejniczak O, Sibiak R, Gutaj P, Wender-Ozegowska E (2023). Pathophysiology of pre-eclampsia-two theories of the development of the disease. International Journal of Molecular Sciences.

[ref-30] Langfelder P, Horvath S (2008). WGCNA: an R package for weighted correlation network analysis. BMC Bioinformatics.

[ref-31] Li C, Hu J, Li M, Fan X, Mao Y (2024a). Integrating machine learning and multi-omics analysis to develop an immune-derived multiple programmed cell death signature for predicting clinical outcomes in gastric cancer. Oncologie.

[ref-32] Li Q, Wei X, Wu F, Qin C, Dong J, Chen C, Lin Y (2024b). Development and validation of preeclampsia predictive models using key genes from bioinformatics and machine learning approaches. Frontiers in Immunology.

[ref-33] Livak KJ, Schmittgen TD (2001). Analysis of relative gene expression data using real-time quantitative PCR and the 2(-Delta Delta C(T)) method. Methods.

[ref-34] Lu X, Wang Y, Geng N, Zou Z, Feng X, Wang Y, Xu Z, Zhang N, Pu J (2024). Dysregulated mitochondrial calcium causes spiral artery remodeling failure in preeclampsia. Hypertension.

[ref-35] MacDonald TM, Walker SP, Hannan NJ, Tong S, Kaitu’u-Lino TJ (2022). Clinical tools and biomarkers to predict preeclampsia. EBioMedicine.

[ref-36] Montgomery KS, Hensley C, Winseman A, Marshall C, Robles A (2024). A systematic review of complications following pre-eclampsia. Maternal and Child Health Journal.

[ref-37] Okina Y, Sato-Matsubara M, Kido Y, Urushima H, Daikoku A, Kadono C, Nakagama Y, Nitahara Y, Hoang TH, Thuy LTT, Matsubara T, Ohtani N, Ikeda K, Yoshizato K, Kawada N (2023). Nitric oxide derived from cytoglobin-deficient hepatic stellate cells causes suppression of cytochrome c oxidase activity in hepatocytes. Antioxidants & Redox Signaling.

[ref-38] Opichka MA, Rappelt MW, Gutterman DD, Grobe JL, McIntosh JJ (2021). Vascular dysfunction in preeclampsia. Cells.

[ref-39] R Core Team (2019). R: a language and environment for statistical computing. https://www.r-project.org.

[ref-40] Roberts JM (2024). Preeclampsia epidemiology(ies) and pathophysiology(ies). Best Practice & Research. Clinical Obstetrics & Gynaecology.

[ref-41] San Juan-Reyes S, Gómez-Oliván LM, Islas-Flores H, Dublán-García O (2020). Oxidative stress in pregnancy complicated by preeclampsia. Archives of Biochemistry and Biophysics.

[ref-42] Schlaepfer IR, Joshi M (2020). CPT1A-mediated fat oxidation, mechanisms, and therapeutic potential. Endocrinology.

[ref-43] Shang Y, Wang X, Su S, Ji F, Shao D, Duan C, Chen T, Liang C, Zhang D, Lu H (2024). Identifying of immune-associated genes for assessing the obesity-associated risk to the offspring in maternal obesity: a bioinformatics and machine learning. CNS Neuroscience & Therapeutics.

[ref-44] Smith AN, Wang X, Thomas DG, Tatum RE, Booz GW, Cunningham MW (2021). The role of mitochondrial dysfunction in preeclampsia: causative factor or collateral damage?. American Journal of Hypertension.

[ref-45] Tayyar AT, Tayyar A, Kozali S, Karakus R, Karakus S, Yuksel IT, Dag I, Yildirim GY, Demirci O (2019). Maternal cytoglobin (CYGB) serum levels in normal and preeclamptic pregnancies. The Journal of Maternal-Fetal & Neonatal Medicine.

[ref-46] Vennou KE, Kontou PI, Braliou GG, Bagos PG (2020). Meta-analysis of gene expression profiles in preeclampsia. Pregnancy Hypertension.

[ref-47] Vishnyakova P, Elchaninov A, Fatkhudinov T, Sukhikh G (2019). Role of the monocyte-macrophage system in normal pregnancy and preeclampsia. International Journal of Molecular Sciences.

[ref-48] Wang H, Li Q, Wang H, Song W (2024). Construction and validation of a line chart for gestational diabetes mellitus based on clinical indicators. Lipids in Health and Disease 2024.

[ref-49] Wang H, Wang Y, Zhong Y, Yu B, Liu D, Jia C, Wu J, Zeng G, Wang Q, Liu F, Sheng C, Huang L (2025). Pasteurized *Akkermansia muciniphila* ameliorates preeclampsia *via* inhibiting mitochondrial dysfunction-mediated placental apoptosis *in vivo* and in vitro. Free Radical Biology & Medicine.

[ref-50] Wu Y, Huang J, Liu L, Zhang X, Zhang W, Li Q (2024). CircHIPK3/miR-124 affects angiogenesis in early-onset preeclampsia *via* CPT1A-mediated fatty acid oxidation. Journal of Molecular Medicine.

[ref-51] Wu Q, Wang L, Wei H, Li B, Yang J, Wang Z, Xu J, Zhou YL, Zhang B (2020). Integration of multiple key molecules in lung adenocarcinoma identifies prognostic and immunotherapeutic relevant gene signatures. International Immunopharmacology.

[ref-52] Wu M, Xu T, Sun H, Gu L, Chu J, Hu P, Zhou W, Feng H, Wang Z, Su F (2025). Single-cell data and network pharmacology screen hub genes related to oxidative stress response in myeloid cell of osteoarthritis. Current Pharmaceutical Analysis.

[ref-53] Yong HE, Melton PE, Johnson MP, Freed KA, Kalionis B, Murthi P, Brennecke SP, Keogh RJ, Moses EK (2015). Genome-wide transcriptome directed pathway analysis of maternal pre-eclampsia susceptibility genes. PLOS ONE.

[ref-54] Yu T, Wang G, Xu X, Yan J (2025). Identification and validation of key biomarkers associated with immune and oxidative stress for preeclampsia by WGCNA and machine learning. Frontiers in Genetics.

[ref-55] Zhang D, Chen X, He X, Deng M, Sun S, Zhu J, Zhang T, Shen S, Zhai X (2026). Multi-omics and machine learning framework reveals ABCG2 as a therapeutic target of Eleven Flavored Shenqi Tablets in clear cell renal cell carcinoma. Journal of Ethnopharmacology.

[ref-56] Zhang X, Chen Y, Sun D, Zhu X, Ying X, Yao Y, Fei W, Zheng C (2022). Emerging pharmacologic interventions for pre-eclampsia treatment. Expert Opinion on Therapeutic Targets.

[ref-57] Zhang B, Liu H, Wu F, Ding Y, Wu J, Lu L, Bajpai AK, Sang M, Wang X (2024). Identification of hub genes and potential molecular mechanisms related to drug sensitivity in acute myeloid leukemia based on machine learning. Frontiers in Pharmacology.

